# Computational systems biology in the big data era

**DOI:** 10.1186/1752-0509-7-S2-S1

**Published:** 2013-10-14

**Authors:** Yong Wang, Xiang-Sun Zhang, Luonan Chen

**Affiliations:** 1 Academy of Mathematics and Systems Science, Chinese Academy of Sciences, Beijing 100190, China; 2 Key Laboratory of Systems Biology, SIBS-Novo Nordisk Translational Research Centre for PreDiabetes, Shanghai Institutes for Biological Sciences, Chinese Academy of Sciences, Shanghai 200031, China

## Abstract

A report of the 6^th ^IEEE International Conference on Systems Biology (IEEE ISB2012), 18-20 August, Xi'an, China.

## Background

According to Wikipedia [1], big data refers to a collection of data sets so large and complex that it becomes difficult to capture, curation, storage, search, sharing, analysis, and visualization. It's well-known that biologists have struggled with 'big data' for a long time. And the situation gets more and more severe. Recently, it is realized that computing power, which doubles roughly every 2 years by Moore's Law, was not sufficient to keep up with the accumulation of sequencing data.

While massive amounts of new sequencing data are being generated worldwide, computing systems biology [2], driven by the complex biological data, should be advancing rapidly enough to digest it. Actually, this energetic interdisciplinary field has kept making significant progresses to mine the biological data to address fundamental questions in biology. Further it is expected to lead to practical applications in medicine, drug discovery, and bio-engineering.

Along with the research development, the societies of computational systems biology are booming too. Our IEEE International Conference on Computational Systems Biology (IEEE ISB), launched six years ago, continues to serve as a high-quality platform and brought many researchers and students to freely exchange ideas [3][4][5]. The 6th IEEE International Conference on Computational Systems Biology (IEEE ISB2012) was successfully organized by Chinese Academy of Sciences and Xidian University. We highly expect that the joint efforts of societies, funding agencies, research institutes, and universities will further push the development of computational methodologies, algorithms, and software in big data era.

## Meeting report

A three-day international conference on Computational and Systems Biology was held in in Xi'an, China, August 18-20. More than 200 researchers including engineers, physicians, mathematicians, and biologists from China mainland, United States, Hong Kong, Taiwan, Japan, Korea enjoyed both academic exchanges and cultural scenes in Xi'an. Different with previous conferences, ISB2012 added the highlight track to invite the authors to present their research progress in recent published paper. Also ISB2012 set up the best paper award to support young researchers.

The Proceedings of the 6th International Conference on Computational Systems Biology (IEEE ISB2012) have been published by IEEE and are available online (http://ieeexplore.ieee.org/xpl/mostRecentIssue.jsp?punumber=6306342). Fifty-seven papers in this volume cover wide range of computational systems biology. Moreover, the reviewers from the Program Committee of IEEE ISB2011 selected 15 papers to be recommended for a special issue in BMC Systems Biology after significant extension of their original versions on the Proceedings. Each submission has been peer reviewed and evaluated by three independent reviewers on the quality, originality, soundness, and significance of its contributions and the significant improvement regarding to the IEEE ISB2012 proceeding paper. Here we focus on some of the highlights of the meeting by categorizing and briefly introducing these selected papers.

## Deep Sequencing data analysis and integration

We are currently generating massive data sets. Especially sequencing data is growing astronomically. New algorithms for data analysis and integrations are in pressing need. In this issue, Vladimir Trifonov et al. noticed that next-generation sequencing technologies have become a major tool for obtaining the difference between the samples. They developed a method, Statistical Algorithm for Variant Frequency Identification (SAVI), to estimate the frequency of alleles in a set of samples from RNA sequencing experiments. Hao Zhang et al. aimed to predict miRNA target from large scale data analysis and developed machine-learning features and conducted comprehensive data training for predicting interactions between H1N1 genome segments and host miRNA. Junhua Zhang et al. tamed the Cancer Genome Atlas (TCGA) glioblastoma multiforme (GBM) and ovarian carcinoma data and proposed a novel method to identify Mutated Core Modules in Cancer without any prior information other than cancer genomic data from patients with tumors. Yangfan Hu et al. performed meta-analysis on different published glioma gene expression profiles and showed an integrated dataset of expression microarrays, microRNA and ChIP-seq profiles representing the significant signatures of different data sets is more similar at pathway level than at gene level. Jingde Bu et al. proposed a two steps heuristic splice alignment tool to deal with RNA-Seq data and can provide both nonconservative and conservative splice junction information. Zhiyuan Yang et al. focused on the recent genome sequencing project of the naked mole rat (NMR, Heterocephalus glaber) and carried out genome-wide comparative analysis of NMR and rat genes. This study provided insights into understanding the possible anti-cancer mechanisms of NMR as well as searching for new cancer-related candidate genes.

## Network systems biology

Network is a way to manage large quantities of biological data by modelling the biological molecules interactions. Biomolecular network concept is well-known as "network biology" and "network medicine" to understand cellular behavior in the systems level in terms of the spatiotemporal interactions among cellular components [6][7]. In this issue, Xinrong Zhou et al. proposed network clustering concepts and computationally investigated the macroscopic changes in the regulatory coordination of diabetes progression in three periods, the early (period of 4 weeks), middle (periods of 8 and 12 weeks) and late (periods of 16 and 20 weeks) stages, in three tissues, adipose, liver and muscle, of Goto-Kakizaki (GK) rats. Xinghuo Ye et al. we defined the significant triple relations among miRNAs, TFs and mRNAs as circuits and investigated the association of transcriptional and post-transcriptional regulating activities in the mouse lung development. Fuhai Li et al. proposed 3D multiscale model and provided a new framework for modeling and simulation studies of cancer stem cell-initiated tumor development. Limin Li et al. proposed two new approaches to identify the metabolic biomarkers with integration of disease specific gene expression data and the genome-scale human metabolic network. Shaoqiang Zhang et al. designed a novel metric, called SPIC (Similarity between Positions with Information Contents), for quantifying the similarity between a column of a motif and a column of another motif. Morihiro Hayashida et al. employed conditional random field (CRFs) to predict the interactions between protein residues and RNA bases. Their study is help to uncover molecular networks and functions in cellular systems

## Software development

Developing software is the typical way for computational biologists to tackle the big data challenges. In this issue, Qiang Huang et al. observed that a lot of biomolecular networks are built from the large scale experimental data produced by the rapidly developing high-throughput techniques as well as literature and other sources. They developed a novel network querying method CNetQ and CNetA, which are implemented in a new R package Corbi (http://doc.aporc.org/wiki/Corbi) and are freely accessible. The computational experiments on the simulated and real data show that their methods get the best accuracy. Huayong Xu et al. released the cGRNB (combinatorial Gene Regulatory Networks Builder): a web server for building combinatorial gene regulatory networks through integrated engineering of seed-matching sequence information and gene expression datasets. The cGRNB enables two major network-building modules, one for MPGE (miRNA-perturbed gene expression) dataset and another for parallel miRNA/mRNA expression datasets. Yue Deng et al. proposed ppiPre, an open-source framework for PPI analysis and prediction using a combination of heterogeneous features including semantic similarities based on GO, co-pathway similarity based on KEGG and similarities based on PPI network topology. ppiPre is implemented in R language and is freely available on the CRAN (http://cran.r-project.org/web/packages/ppiPre/).

## Competing interests

The authors declare that they have no competing interests.

## Authors' contributions

YW drafted the manuscript. XSZ and LC read and approved the manuscript.
